# Reconstructing Druze population history

**DOI:** 10.1038/srep35837

**Published:** 2016-11-16

**Authors:** Scarlett Marshall, Ranajit Das, Mehdi Pirooznia, Eran Elhaik

**Affiliations:** 1 Independent Researcher, Oxford, UK; 2 Manipal Centre for Natural Sciences (MCNS), Manipal University, Manipal, Karnataka, India; 3 Johns Hopkins University, Department of Psychiatry and Behavioral Sciences, Baltimore, MD 21205, USA; 4 University of Sheffield, Department of Animal and Plant Sciences, Sheffield S10 2TN, UK

## Abstract

The Druze are an aggregate of communities in the Levant and Near East living almost exclusively in the mountains of Syria, Lebanon and Israel whose ~1000 year old religion formally opposes mixed marriages and conversions. Despite increasing interest in genetics of the population structure of the Druze, their population history remains unknown. We investigated the genetic relationships between Israeli Druze and both modern and ancient populations. We evaluated our findings in light of three hypotheses purporting to explain Druze history that posit Arabian, Persian or mixed Near Eastern-Levantine roots. The biogeographical analysis localised proto-Druze to the mountainous regions of southeastern Turkey, northern Iraq and southeast Syria and their descendants clustered along a trajectory between these two regions. The mixed Near Eastern–Middle Eastern localisation of the Druze, shown using both modern and ancient DNA data, is distinct from that of neighbouring Syrians, Palestinians and most of the Lebanese, who exhibit a high affinity to the Levant. Druze biogeographic affinity, migration patterns, time of emergence and genetic similarity to Near Eastern populations are highly suggestive of Armenian-Turkish ancestries for the proto-Druze.

The population history of the Druze people, who accepted Druzism around the 11^th^ century A.D., remains a fascinating question in history, cultural anthropology and genetics. Contemporary Druze comprise an aggregate of Levantine and Near Eastern communities residing almost exclusively in the mountain regions of Syria (500,000), Lebanon (215,000), Israel (136,000) and Jordan (20,000), although with an increasingly large diaspora in the USA^1^,^2^,^3^. Almost half of the total population of Druze live on Mount Hauran, alternatively known as Jabal al-Durūz, or the Mountain of the Druze, south of Damascus. The remainder live in the Lebanese Mounts Lebanon and Shuf and the Israeli Mount Carmel, Golan Heights and the Upper Galilee region. Shortly after its modest beginnings in the early 11^th^ century A.D., Druzism garnered rapid support in the Levant^4^; and its adherents, albeit small in number, have played a disproportionately large role in the social, political and cultural shaping of the Levant (and Israel in particular^5^) making the question of their history even more intriguing.

This question is particularly challenging given the Druze’s original nomadic lifestyle and the development, over time, of their esoteric religion that incorporates Isma’ilism Islam, Judaism, Zoroastrian, Hindu, Christian, Neo-Platonic and Persian influences^4^. Although very little is known of the religion itself, since the actual practices and the scriptures, tenets and beliefs are zealously guarded, its conceptual montage is highly suggestive of a diverse origin^6^.

Druzism was first reported in Cairo under the sixth caliph of the Fatimid Dynasty, Al-Hakim (996–1021 A.D.) who sent missionaries throughout Arabia and the Middle East (with epistles being recorded as far as India), calling for new adherents to join the religion^7^. After Al-Hakim’s disappearance, the new caliph, Alī az-Zāhir (1021–1036 A.D.) ruthlessly oppressed Druze followers and effectively eradicated Druzism in Cairo^8^. Any surviving Druze are believed to have fled to neighbouring mountains^9^. Later on, proselytisation efforts ceased, intermarriage between Druze and non-Druze was prohibited and the religion took on a new form, concealed from outsiders. By this time, Druzism had taken root in north Palestine with the earliest Druze communities recorded around Mount Hermon^4^. Mount Hermon has remained a key location for Druze communities throughout their history, although we cannot be certain that the contemporary Druze studied here have descended from Middle Age Druze.

Several hypotheses have been proposed to explain the history of contemporary Druze based on historic, archaeological and linguistic evidence (Table 1). These hypotheses espouse that Druze emerged from Arabian tribes such as the Tanukhs or Itureans, from Persian populations (a theory grounded in the lexical similarities between Persian and Druze texts^4^) and from mixed Near Eastern-Levantine populations, which would relate Druze to migratory Near Eastern and Syrian tribes who accepted the faith around the 11^th^ century A.D. However, none of these hypotheses are supported by incontestable evidence leaving the population history of the Druze unresolved. This study aims to employ genetic tools to generate a more comprehensive history of Druzism.

The work of Cavalli-Sforza and other investigators has already established the strong relationship between geography and genetics^10^,^11^. This parallelism allows inferring the geographic regions where populations with a population structure similar to that of the Druze reside. High similarity typically implies shared genetic ancestry with these populations, assuming that the primeval population structure of the Druze has been largely preserved through endogamous practices and limited conversions. This approach has been widely used in previous genetic studies, focusing on uniparental chromosomes. These studies reported that the Galilee Druze exhibit a high diversity of both X1 (15.6%) and X2 (11.1%) mitochondrial haplogroups relative to other Near Eastern populations^12^,^13^,^14^, however they were based on small sample sizes. The Druze were reported to exhibit up to a 300-fold difference in haplogroup frequencies between these studies^12^,^13^ and up to a 1600-fold difference when compared to other Israeli sites^12^. More recent studies have reported that the Druze share many genetic characteristics with other Levantine populations (i.e., Palestinians and Bedouins) compared to Europeans and Central or South Asians e.g.^15^,^16^,^17^,^18^. However, these studies have focused on the ancestry of the Druze, relying mostly on principle component (PC) and structure-like analyses, which have major limitations^19^,^20^ and are unsuitable to biogeography^10^. Diffused Southern Turkish and Northern Syrian origins were recently proposed for the Druze using a PC-based application for biogeography^21^.

Due to their unknown population history, remote living and the theoretical prohibition of marriage with non-Druze, the Druze have been characterised by some authors as a ‘population isolate,’ ‘genetic isolate’ and a ‘genetic refugium’ e.g.^12^,^22^, making them attractive candidates for epidemiological studies. Although such inferences cannot be drawn based on a *presumed* social structure and geographical preference, genetic efforts targeting the Druze have identified several rare, mainly monogenic, recessive disorders like Behçet’s disease^23^ in several Druze individuals.

To improve our understanding of the population history of Israeli Druze, we performed genome-wide and haplogroup analyses, the results of which were compared with Levantine and non-Levantine populations. Our findings are evaluated in light of the three major hypotheses depicting Saudi Arabian, Near Eastern and Iranian-Iraqi roots for Druze (Table 1), though these may not be mutually exclusive. Due to changes in the population structure of Druze over the past millennia, we do not expect that our biogeographical predictions will perfectly agree with the predictions made by any one hypothesis.

## Results

The rationale of biogeographical analyses is that the population structure of the population of interest, and that of its parental populations, would exhibit high similarity allowing us to infer the biogeographical affinity of the population in question from the known geographical location of the parental populations^24^. Our search for the region affiliated with the population structure of Druze focused on North Africa, Asia and Eastern Europe covering the biogeographical affinity predicted by each hypothesis (Table 1). All biogeographical inferences were carried out using the geographic population structure (GPS) tool^10^. Briefly, GPS accepts the DNA of individuals and matches their admixture proportions to those of reference populations known to have resided in a certain geographical region for a substantial period of time. GPS then converts the genetic distances to geographic distances. A population movement followed by gene exchanges with other populations modifies the admixture signature, while isolation and segregation preserve the original admixture signature of the migratory population. Therefore, GPS predictions correspond to the last place in which significant admixture has occurred in relation to the reference populations, termed here *biogeographical affinity*. For individuals of mixed ancestries, GPS coordinates represent the mean geographical locations of their immediate parental populations^25^.

### Biogeographical mapping of Afro-Eurasian population

**Figure 1:**
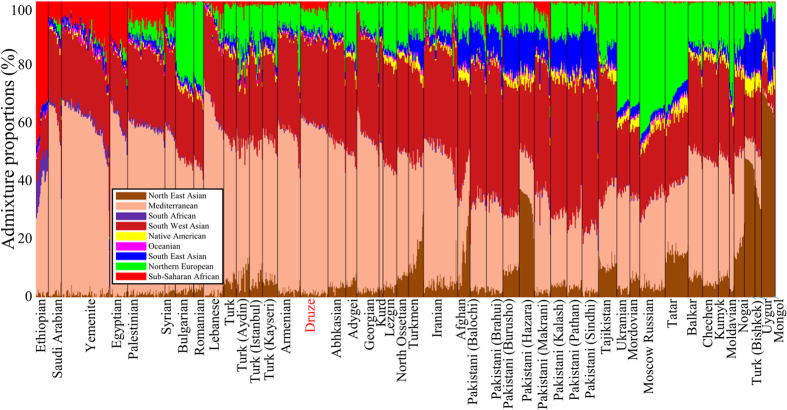
Admixture proportions of all populations included in this study. For brevity, subpopulations were collapsed. The x axis represents individuals. Each individual is represented by a vertical stacked column of colour-coded admixture proportions that reflects genetic contributions from nine putative ancestral populations.

Prior to applying GPS to elucidate the biogeographical affinity of Druze, we sought to trial its accuracy on Afro-Eurasian populations. For that, we analysed over 1,000 individuals belonging to 50 Afro-Eurasian populations and estimated their admixture proportion in respect to nine admixture components corresponding to putative ancestral populations (Fig. 1). All the genomes consist of at least four admixture components segregating within and between neighbouring populations. In western Eurasians the most dominant components are Mediterranean, Southwest Asian and Northern European, with the latter taking over the Sub-Saharan component in Eurasia. Genetic diversity was estimated by computing the genetic distances (*d*) defined as the minimal Euclidean distances between the admixture proportions of each individual and all members of a population of interest. Small genetic distances indicate high genetic similarity. The median genetic distances for all populations (Figure S1) were relatively small, ranging from 1.6% to 10.2% with a mean of 2.7%.

**Figure 2:**
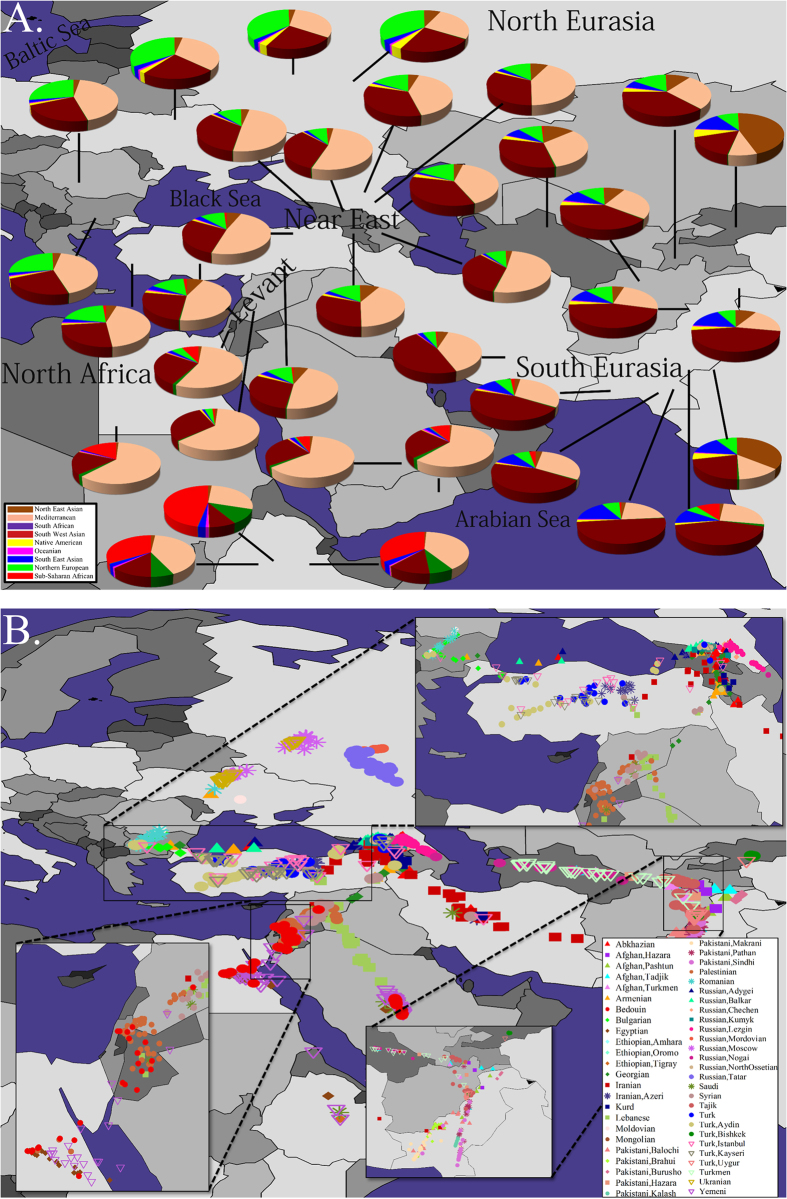
GPS predictions of biogeographical affinities for Afro-Eurasian individuals. (**A**) Pie charts reflect the admixture proportions and geographical locations of each reference population excluding reference subpopulations, for brevity. (**B**) GPS predicted coordinates for Afro-Eurasian populations and subpopulations. Insets show the results in higher resolution. *Note*: occasionally all individuals of certain populations were predicted to the same spot and thus appear as a single individual. All maps were plotted using the R package rworldmap (Ver 1.3-1, https://r-forge.r-project.org/R/?group_id=1497)^74^.

We applied GPS using the l*eave-one-out* procedure at the population level. Assignment accuracy was determined for each individual based on whether the predicted geographical coordinates were within 500 kilometres (km) or 250 km of the political boundaries of their country or regional location. GPS correctly assigned 77% and 68% of the individuals to less than 500 km and 250 km from their countries, respectively (Fig. 2, Table S2). These results illustrate the strong genomic-geographic relationship and demarcate the expected assignment error for the Druze.

### Biogeographical mapping of the Druze

Like most Eurasians, the Druze genomes exhibit a mixture of three major components: Mediterranean (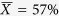), Southwest Asian (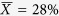) and Northern European (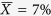) (Fig. 1). Druze and Syrians possess a significantly larger amount of the Northern European component (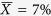) when compared with their neighbouring populations, such as Palestinians (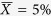) and Lebanese and Bedouins (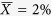) (Kolmogorov-Smirnov goodness-of-fit tests, [Syrians] *p*-value < 0.05, [Palestinians] *p*-value < 10^−10^, [Lebanese and Bedouins] *p*-value < 10^−11^). Using Alder^26^ we found that there is evidence for admixture in Druze between the early 9^th^ century and the early 12^th^ century (38.62 ± 5.8 generations ago). This estimate is within the range proposed by two recent studies using the less accurate ROLLOFF^26^ (46 ± 5)^18^ and CHROMOPAINTER (31.64 ± 4 generations ago)^27^.

**Figure 3:**
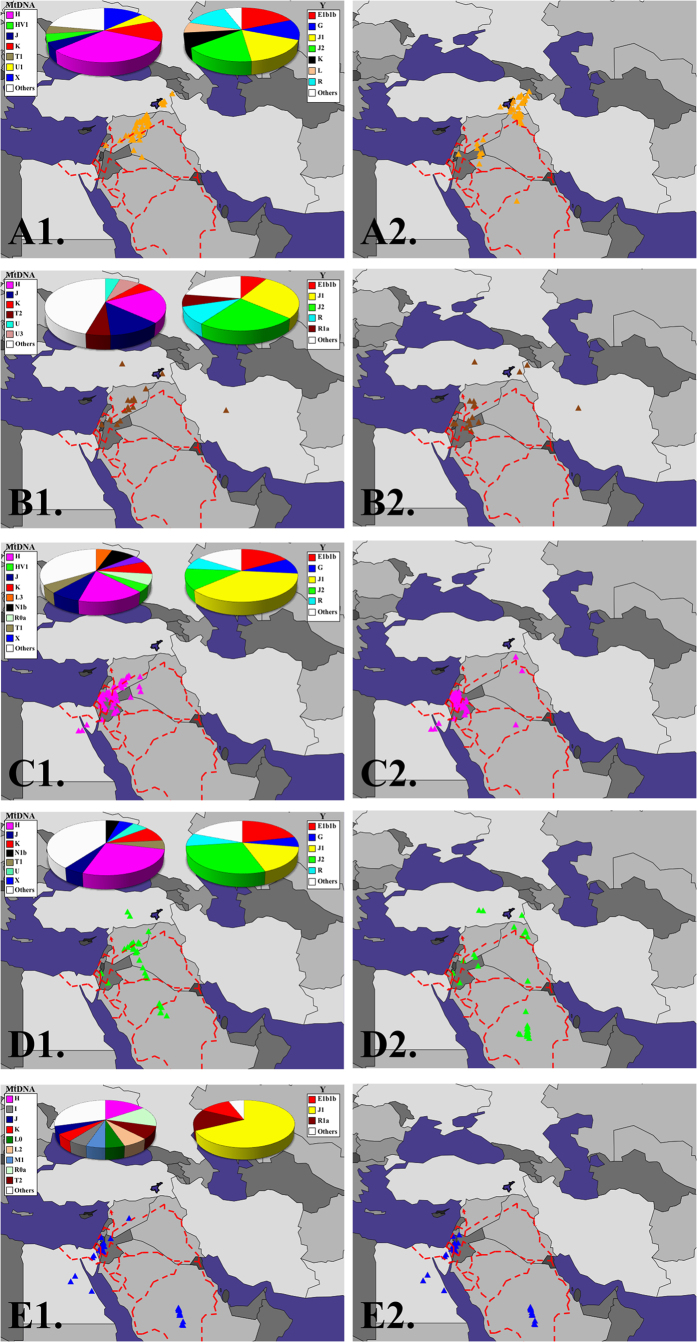
GPS results for Levantine populations. A map depicting the GPS predicted locations for Druze (**A1**), Syrians (**B1**), Palestinians (**C1**), Lebanese (**D1**) and Bedouins (**E1**). The paternal and maternal haplogroups (e.g., mtDNA haplogroup H) of each population are shown at the top of the figures and listed in the legend. Maps depicting GPS-predicted locations after excluding Syrians from the reference panel are marked as 2 (e.g., **A2**). Dashed lines indicate the Nabataean caravan and trade routes. All maps were plotted using the R package rworldmap (Ver 1.3-1, https://r-forge.r-project.org/R/?group_id=1497)^74^.

**Figure 4:**
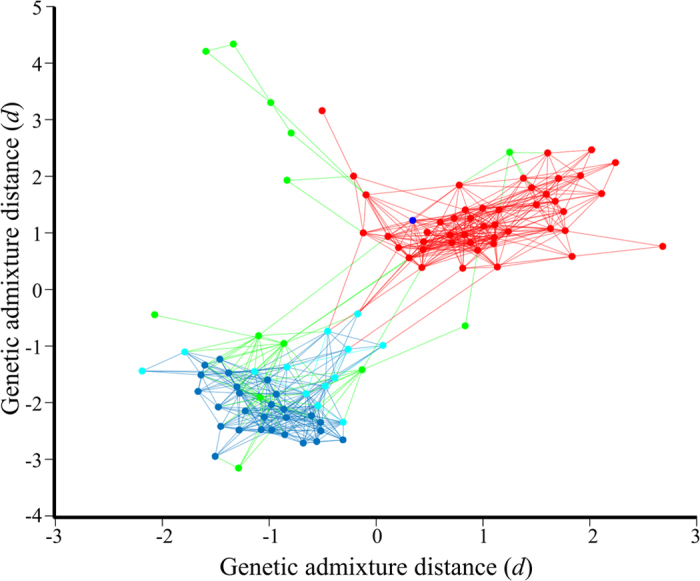
Undirected graph illustrating the genetic distances between Druze, Syrians and Palestinians. For coherency, edges are shown only between individuals whose genetic admixture distances are smaller than 0.3. Druze individuals clustered with Syrians (cyan) or Palestinians (dark blue) were highlighted. As expected, this analysis indicated that the Druze are closest to Syrians, particularly those predicted by GPS to originate in Syria. Bedouins clustered northeast to the Palestinians and were excluded from this plot for brevity.

We next applied GPS to infer the biogeographical affinity of the Druze using all reference populations. GPS positioned nearly all 42 Druze along a trajectory going from the Armenian-Turkish border to Syria (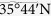, 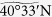) (Fig. 3[A1]) with 17% of the individuals localised to the mountainous region surrounding Lake Van. This prediction is in concordance with the location (38°36 ± 3°45′*N*, 36°25 ± 1°41′*E*) obtained using a PCA-based application for biogeography, suggesting a diffused Southern Turkish and Northern Syrian affinity for the Druze^21^. These results are highly surprising for a population depicted by some authors as genetically isolated, in which case they would have been expected to cluster tightly in a single region. To uncover the more primeval biogeographical affinity of these individuals, we removed the Syrian subpopulations to which most Druze adhered, from the reference panel and repeated the analysis so that Druze predictions would be affected by their secondary closest populations. This split Druze into two major subpopulations some 700 km apart from one other; the larger one (79% of the Druze) centred around the mountainous regions of the Turkish Hakkari and Van Provinces as well as northern Iraq (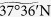, 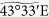), close to Mounts Artos and Ararat and the second one in southeast Syria (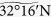, 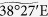) with a median distance of 168.6 kilometres (km) from Mount Hauran (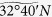, 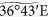), formerly known as the State of Jabal Druze, where nearly half a million Druze reside today (Fig. 3[A2]). None of the Druze were localised in Israel. A comparison of the genetic distances between the Druze and the reference populations (Figure S2) confirmed that Druze individuals exhibit the shortest genetic distances to Syrians (31%), Palestinians (31%) and Lebanese (24%) followed by Armenians (12%) and Saudis (2%). To illustrate the genetic distances between Levantine individuals, we plotted the genetic distances between Druze, Syrians and Palestinians (Fig. 4). Surprisingly, we found a Druze individual (HGDP00576) whose admixture signature resembled a Palestinian one, likely due to a very recent gene exchange event.

### Biogeographical mapping of Levant populations

To test whether Druze and their neighbouring populations share biogeographical affinities, we repeated the GPS analyses for 16 Syrians, 57 Palestinians, 31 Lebanese and 45 Bedouins (Fig. 3). GPS localised Syrians to Syria (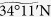, 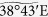) even after excluding the Syrian from the reference population panel (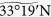, 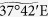) (Fig. 3[B1,B2]). Only 6–12.5% of the Syrians were localised to the Lake Van region (Fig. 3[B1,B2]). The Palestinians were also highly localised to North Israel, West Jordan and Syria (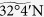, 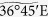) (Fig. 3[C1,C2]). While 36% of the Lebanese clustered in Syria, most of the Lebanese (45%) were localised along a trajectory parallel to the Incense Route leading from South Arabia to the Mediterranean (Fig. 3[D1]). Excluding Syrians, their closest population, supported a primarily Arabian root for the Lebanese and secondarily Syrian (Fig. 3[D2]). None of the Lebanese clustered close to Lake Van with only 6% in central Turkey. The Bedouins clustered in Israel, Jordan, Egypt and Saudi Arabia, as would be expected from their pastoral and nomadic history (Fig. 3[E1,E2]).

### Ancient DNA analyses

**Figure 5:**
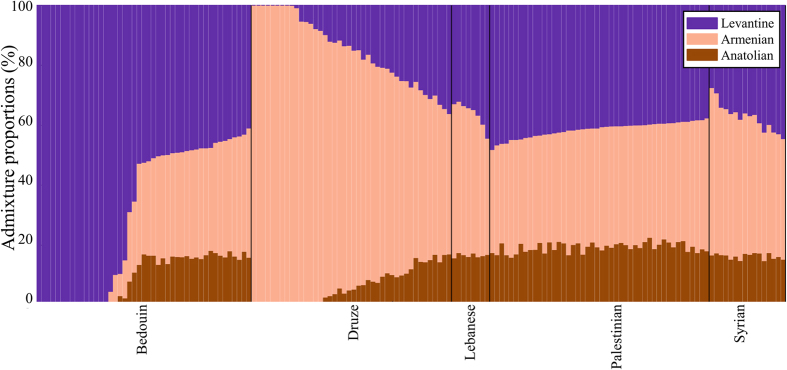
Ancient admixture proportions of Druze and Levantine populations. For brevity, subpopulations were collapsed. The x axis represents individuals. Each individual is represented by a vertical stacked column of colour-coded admixture proportions that reflects genetic contributions from ancient Levantine, Armenian and Anatolian individuals.

Our biogeographical analyses highlight the high genetic similarity between Druze and Near Eastern populations, compared with Levantine populations who were predicted close to each other and some of the Druze. This is in agreement with an ancient DNA study^28^ where Druze clustered with few Lebanese, close to a Chalcolithic Anatolian and Chalcolithic/Bronze Age Armenians, and away from Levantine populations who clustered with Neolithic/Bronze Age Levantines. To obtain further insights into the population structure of Druze and Levantine populations in relation to ancient populations, we carried out a supervised admixture analysis using ancient Levantine, Armenian, and Anatolian individuals dating to the Neolithic, Chalcolithic and Bronze Age periods as ancestral populations. A third of the Druze and Bedouins show complete Armenian and Levantine ancestries, respectively. The remaining genomes exhibit a mixture of the three ancestries (Fig. 5). Interestingly, Druze possess a significantly larger amount of ancient Armenian ancestry (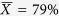) compared to other Levantine populations (21.12% < 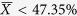) (Kolmogorov-Smirnov goodness-of-fit tests, [modern-day Levantine populations] *p*-value < 10^−25^). Druze also have a significantly smaller ancient Levantine ancestry (14.9%) compared to other Levantine populations (36.07% < 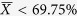) (Kolmogorov-Smirnov goodness-of-fit tests, [modern-day Levantine populations] *p*-value <10^−25^). The ancient Anatolian component distributes nearly evenly (18–22%) among mixed Levantine individuals and exhibits a gradient in Druze (0–17%). These results are in agreement with our findings using modern-day populations that indicated a high similarity between Druze and Near Eastern populations over Levantine populations and the complex population structure of the Druze (Fig. 3[A2]), which is distinct from that of Levantine populations (Fig. 5).

### Haplogroup analyses

The Druze belong to nearly all the basal Y chromosomal and mtDNA haplogroups (Tables S4 and S5, respectively). The most common mtDNA haplogroups (H, K, U1, X and J) are present in 68.76% of the individuals compared with 94.17% of the individuals that exhibit the most common Y haplogroups (E1b1b, G, J1, J2, K, L and R [except R1a]). Maternal haplogroups H, K and X are significantly more common in Druze (31.88%, 12.81% and 12.81%, respectively) than in all other Levantine populations (15.52–26.74%, 5.17–8.91% and 0.61–4.74%, respectively) (Fisher’s exact-test, *p*-value 0.0002, 0.0027, and <0.0001, respectively). However, the K and H haplogroups are not significantly higher in Druze (12.81% and 31.88%, respectively) compared to Lebanese (8.91% and 26.74%, respectively) (Fisher’s exact-test, *p*-values 0.08 and 0.07 respectively). There is no significant difference in the maternal haplogroup U between Druze (5.63%) and other Levantine populations (3.06–3.66%) (Fisher’s exact test, *p*-value = 0.059). There is also no significant difference in maternal haplogroup J between Druze (5.63%) and Levantine populations (7.55%) (Fisher’s exact test, *p*-value = 0.15). However, compared to the Syrian population (12.05%) the J haplogroup count is significantly lower in Druze (Fisher’s exact test, *p*-value = 0.0033). The combined frequency of the K and L (14.58%) Y haplogroups in Druze is much higher than in other Levant populations (3.6–4.6%) (Fisher’s exact-test, *p*-value < 0.0001), whereas the Levantine frequencies of J1 and J2 (45–67.6%) are lower in Druze (33.53%) (Fisher’s exact-test, *p*-value < 0.0001) and higher in Bedouins (67.6%).

The Druze’s most common mtDNA haplogroups explain a much higher fraction of the variation compared to other Levant populations (32.76–48.19%) as has been previously observed^12^, whereas their Y haplogroups explain a higher but comparable proportion of the variation (79.28–88.71%). The high haplogroup variability between sampling sites^12^ can be explained by our relatively small sample size. Due to the high levels of endogamy practiced among Druze, lineages tend to cluster in villages and in non-random patterns which can obscure the haplotype diversity in the entire population. It is therefore necessary to test a much larger number of individuals from various areas to obtain an accurate description of their Y chromosomal diversity.

The most common Y haplogroups in Druze dominate the area between the Black and Caspian Seas and represent the major lineages among populations inhabiting Western Asian regions, including Turkey, Iran, Afghanistan and the Caucasus^29^,^30^,^31^. The mtDNA haplogroups also indicate a Eurasian origin due to the commonality of the haplogroups in Central Asia (e.g., J), Europe (e.g., H), North Eurasia (e.g., T) and Northeast Eurasia (e.g., X)^32^.

## Discussion

The present study aims to shed light on the population history of the Israeli Druze and assess the findings in light of three hypotheses advocating Arabian, Iranian-Iraqi or Near Eastern roots (Table 1) while examining claims that the Druze are a ‘genetic isolate’. Considering the Druze admixture components, biogeographical affinity, paternal and maternal haplogroups and genetic similarity to neighbouring populations allows us to tentatively reconstruct their history and explain some of their habitual preferences.

### Evaluating the evidence for the biogeographical affinity of Druze

Although predicted in part to Syria (Fig. 3[A1]), like Palestinians, Lebanese and Syrians (Fig. 3[B–D]), only a minority of the Druze (Fig. 3[A2]) could be considered to be highly localised to the Levantine. The mixed population structure of the Druze has two biogeographical affinities: a southeast Turkish-northern Iraqi one overlapping the Zagros Mountains and close to Mount Ararat and a southeast Syrian one, close to Mount Hauran. Though the Turkish affinity of the Druze can be observed for a smaller fraction of Druze, likely due to on-going gene exchange with Syrians (Fig. 3[A1]), it can still be recognised as the primary affinity of nearly 80% of Druze (Fig. 3[A2]), suggesting its antiquity compared to the Syrian affinity. Such a conclusion is in agreement with our ancient DNA analysis since, in relation to ancient individuals (12,000–1000 B.C.), a third of the Druze appear like ancient Armenians, whereas the remaining exhibit nearly 80% ancient Armenian ancestry compared to less than 15% ancient Levantine ancestry (Fig. 5). Altogether these findings suggest that the proto-Druze were from tribes who resided around the Zagros and surrounding mountains and Syrian tribes with whom they exchanged genes (Fig. 3[A1]) subsequent to, and after, their migration to Palestine. We speculate that the gene exchange events with non-Druze were uneven across the population, which helped retain some of the Near Eastern admixture signature that distinguishes Druze from other Levantine populations (Figs 3[A2–E2] and 5). Consequently, the majority of Druze are genetically closer to Syrians than to other Levantine populations (Fig. 4) and share a genetic similarity with Arabians as well as Near Eastern populations (Figure S2). These results are in agreement with those of Elhaik^21^, reporting Southern Turkish and Northern Syrian biogeographical affinities in support of the Near Eastern hypothesis for the emergence of Druze over alternative hypotheses that fail to explain the mixed biogeographical affinities of the Druze (Figs 3 and 4).

### Evaluating the evidence for the biogeographical affinity of non-Druze Levantine populations

The biogeographical affinities of the Druze are unique compared with neighbouring Levantine populations. Only a minute fraction of Lebanese and Syrians share a Turkish affinity (Fig. 3), and both Syrians and Palestinians are highly localised to the Levant. While these results do not rule out a partial Turkish ancestry for some of the Syrians, they suggest that any genetic evidence for such an ancestry has decayed over time due to on-going gene exchange with Levantine populations and the absence of large inflows of migrants with relatively distinct population structure. The biogeographical affinity of Palestinians concurs with previous studies employing uniparental markers^33^ and historical records, which suggest that they descended, at least in part, from local Israelite inhabitants who converted to Islam following the Muslim conquest in the early 7^th^ century^6^,^34^.

Fascinatingly, most Lebanese individuals were predicted along the northwestern Incense Route leading from southern Arabia to the Mediterranean, used by merchants between the 4^th^ century B.C. and the 2^nd^ century A.D. This multi-origin of the Lebanese (Fig. 3[C1]) may be explained either by the 7^th^ century Arabian expansion, which saw a large scale movement of Arabian tribes from the Arabian Peninsula into the Middle East, or by the northern expansion of nomadic Bedouin tribes known as the Nabataeans. By the end of the fourth century the Nabataeans had established an empire which occupied Northern Arabia and the Southern Levant for four hundred years, making migration into Lebanon at this time highly probable^35^. However, as both Nabatean and late Arab conquerors inhabited the same geographical regions and emerged around similar historical periods, they likely share the same genetic background. Therefore, the exact ancestry of Lebanese cannot be properly deciphered without ancient DNA from the potential ancestral populations, currently unavailable.

### Reconstruction of Druze population history

When combined with historical and anthropological records, our findings allow a cautious reconstruction of certain aspects of Druze population history. First recorded as “mountain dwellers” as early as the 12^th^ century A.D.^4^, the Druze exhibit a consistent propensity for residing in the highest mountains whether in Israel (Mounts Hermon and Carmel), Syria (Mount Hauran) or Lebanon (Mounts Lebanon and Shuf)^5^. These mountains provide the Druze with protection and allow them to maintain the close societal structure that is integral to their religious practices. This critical aspect of Druze life has been neglected by many previous studies on the origin of Druze. Our GPS analyses localised most Druze to the highest and largest mountainous Regions of southeast Turkey and northern Iraq and the remaining individuals close to the Syrian Mount Hauran, where most Druze reside today. Our analyses also indicated an on-going mixture between these two groups. These findings hint at a tantalizing possibility that, over time, at least some of the proto-Druze may have developed a genetic adaptation to high altitudes, such as has been reported in several other mountainous populations^36^. Our findings are in agreement with the results obtained by *fineSTRUCTURE* where populations were clustered into clades based on their population structure similarity^27^. Druze were clustered into the “West Asian” clade together with Adygei, Armenian, Cypriot, Georgian, Iranian, Lezgin and Turkish populations. Such findings are also in agreement with a recent ancient DNA study^28^, where Druze exhibited genetic similarity to Chalcolithic and Bronze Age Armenians and a Chalcolithic Anatolian. In that study, Druze clustered remotely from all Bronze Age and Neolithic Levantines, whereas Palestinians, Bedouins, Syrians and a few Lebanese clustered with Levantine populations.

The most parsimonious explanation for our findings is that some of the proto-Druze emerged from Armenian-Turkish tribes residing in the Zagros and surrounding mountains, prior to the end of the first millennium A.D. (Figs 3[A1,A2] and 5). It is unclear when these tribes migrated to the Levant, as there have been several small migrations of Turkish people into the region throughout the Middle Ages, and only some of these have left a detectable DNA hallmark^27^. However, the most significant Turkish migration was the expansion of the Seljuk Turkish Empire into the region in the years following the Battle of Manzikert, north of Lake Van (1071 A.D.). By 1079 A.D., the Seljuqs had reached Syria and Palestine and settled in Iran, Anatolia and Syria^37^. The Druze were first recorded in that region ~150 years later^4^. It is therefore possible that the proto-Druze population was part of this early Seljuk expansion. This explanation is supported by the short genetic distances found between the Druze and several Near Eastern populations reported here (Figure S2) and elsewhere^12^ and ancient DNA evidence indicating that this similarity has roots in the Chalcolithic and Bronze Age^28^. During their residence in Syria, and prior to or during, their admission into Druzism, these migratory tribes have probably experienced uneven gene exchanges with Syrians and Lebanese or Arabian tribes dwelling along the Incense route (Fig. 3[A1]), which increased their genetic diversity. Yet we venture that they retained some of their habitual preferences and continued residing in mountains.

Such a scenario, however, may be at odds with accounts of the official closing of the religion to new adherents in 1043 A.D., thirty years prior to the Seljuk expansion^38^. To resolve this contradiction, we speculate that the sealing of Druzism did not necessarily mark the *de facto* sealing of the faith, nor its closure to Middle Eastern proselytes. Although not actively encouraged by religious authorities, old and modern historical records, along with our genetic findings suggest that it is very likely that some conversions to the Druze faith were allowed after the 11^th^ century A.D. For example, Betts^8^ alludes to several notable instances over the past millennium where non-Druze have been admitted into the religion; such as the Jumblatt family, one of the leading Druze political clans of Lebanon. Furthermore, our dating analysis suggests that the major gene exchanges that shaped the Druze genome continued at least until the early 12^th^ century A.D. Since other Levantine populations who do not live in seclusion have similar admixture dates, the admixture date cannot be interpreted as evidence that gene exchanges with neighbouring populations stopped, but rather that no population-wide admixture event with a population that is genetically relatively dissimilar to Levantine populations had occurred^27^.

By the 10^th^ century A.D., the Fatimid dynasty ruled Syria, Lebanon, Palestine, Jordan, Egypt and North Africa, which afforded Al-Hakim, the sixth Fatimid caliph and one of the founders of Druzism, the opportunity to spread his ideas throughout the Middle East. Adherents could have developed their own division of Druzism that incorporated both the original beliefs of Cairo Druze and other early monotheistic religious and philosophical ideas with those which they were previously familiar, including those that may have allowed conversions. Indeed, the Druze religion incorporates eclectic fundamental religious ideas from throughout the Middle East^5^, and the Druze themselves proclaim a diverse descent from Yemenite, Tanukh, Kurdish and Iranian tribes^4^,^35^. Such a heterogeneous Middle Eastern descent is supported by their high haplogroup diversity compared to neighbouring populations (Fig. 3[A1–E1]).

It is therefore not unreasonable to consider that Druze proselytization in Middle Eastern communities endured after the oppression of the Cairo sect. Conversion efforts may have continued on a small scale until such regional operations drew the unwanted attention of local governments, forcing Druze leaders to halt further conversion efforts^5^.

The matter of gene exchange between Druze and non-Druze should be addressed with caution, as marriage to a non-Druze can lead to ostracism from the community^39^ and is still considered a fundamental characteristic of Druze identity^40^ in the Middle East and the diaspora^1^. In 2002, a survey of the Israeli Central Bureau of Statistics reported that the proportion of atheists among Israeli Druze is the highest of all Israelis (48%), including Jews (44%), Arabs (18%), Muslims (12%), and Christian Arabs (35%)^41^. An independent study examined the 145 officially recorded cases of Israeli Druze ‘straying’ from the religion, often motivated in part by a desire to marry outside the community^39^. Despite the excommunication fears, there has been a growing practice of exogamous marriages amongst Druze, particularly in the United States^42^ where inter-religious marriages, especially between Druze men and non-Druze women, are becoming more commonplace. These are becoming increasingly widespread in Israel^43^. However, such practices are expected to change according to the regional marriage laws that may be very strict. For example, in Lebanon, civil marriages are not allowed^44^, whereas in the US fewer prohibitions on marriage exist. With a lack of updated information regarding Druze marital practices, it is reasonable to conclude that the practice of exogamy is on the rise among the Druze, although it is difficult to assess whether this also entails a decline in the number of “religious” Druze due to the changing nature of this term. Secularisation processes, including the decline of strict religious practices such as endogamy^5^, especially among the younger generation^40^, can be expected to intensify the gene exchanges with neighbouring populations over time. For instance, our admixture analysis (Fig. 4) singled out a Druze individual whose admixture signature closely resembles a Palestinian one, probably due to a very recent gene exchange event. Though it may appear insignificant, we note that this individual was found in the HDGP cohort, carefully curated and then analysed thousands of times.

### Misconception of genetic isolates

Evaluating whether Druze are a ‘genetic isolate’ necessitates an understanding of this concept. An ideal genetically isolated population is an endogamous group dating back to ancient times that derived from a small number of individuals who became isolated after a founding event. Such communities would be characterised by minimal mixing and reduced gene flow with neighbouring populations or their potential progenitors, facilitated by strict societal practices, effective geographical barriers or both^45^. Following Cann’s definition, isolated populations are expected to exhibit a small effective population size (*Ne*) in the range of 10–100 individuals (<80 for the New World founding population)^46^,^47^, highly homogeneous genomes in terms of allele frequencies, high inbreeding coefficients and longer runs of homozygosity compared with panmictic populations^32^. Sufficiently long isolation, which has persisted for hundreds of generations, may also generate novel combinations of alleles that could contribute towards otherwise rare genetic disorders becoming more prevalent in the isolated population^46^. A conclusion of genetic isolation can therefore be reached only after extensive genetic comparisons of the putative isolated population with its neighbouring populations and potential progenitors and after excluding artefacts that may lead to such an impression, like small sample sizes, studying insufficient numbers of markers and questionable study designs.

In reality, we estimate that less than 20% of the worldwide populations (estimated at 6,000 populations^48^) have been fully genetically tested, which raises concerns about whether claims of genetic isolation have been sufficiently substantiated. Moreover, in practice, most human populations living inland are not ideal ‘population isolates’ since they have never lived in true isolation nor seclusion. Studies of the apportionment of human genetic variation have long established that most human variation is within population groups and that the additional variation between population groups is small but greatest when comparing different continental populations^49^. The number of actual genetic isolates or even relative isolates is therefore likely to be far lower than the number of populations professed to be so (e.g.^50^,^51^).

Some authors have considered the Druze to be a ‘population isolate’ and a ‘genetic refugium’ based on little or dubious genetic evidence, which fails to meet the above criteria. For example, Shlush *et al.*^12^ stated that the “social structure has turned the Druze into transnational isolates – a population which remains genetically isolated largely through the social practice of endogamy and consanguinity.” The authors also argued that the relatively high frequency of mtDNA X, H and K haplogroups are indicative of isolation (“the refugium hypothesis based on mtDNA haplogroup X analysis was corroborated by the finding of high diversity for the Druze mtDNA haplogroups H and K, with the added finding of novel lineages not shared with nearby populations.”). However, we found that both H and K maternal haplogroups show similar frequencies in Druze and Lebanese (Table S5) and that the X haplogroup variation occurs largely between villages^12^,^52^, which could be a product of genetic drift. Likewise, the lack of paternal haplogroup K in neighbouring populations was misinterpreted as evidence of isolation (“The finding of the enrichment of the [non-recombining Y] NRY haplogroup K among the Galilee Druze with no detection in samples from other subregions, further supports the relative isolation of this region, even among the Druze”), although it is also found among Palestinians (4.6%), Syrians (3.2%) and Lebanese (0.3%) (Table S4). Since variation in haplogroup frequencies is typical between and within human groups, variation in haplogroups alone cannot be taken as an incontestable indicator of genetic isolation. Higher rates of endogamy among Druze are likely to increase the frequency of certain haplogroups through genetic drift.

**Figure 6:**
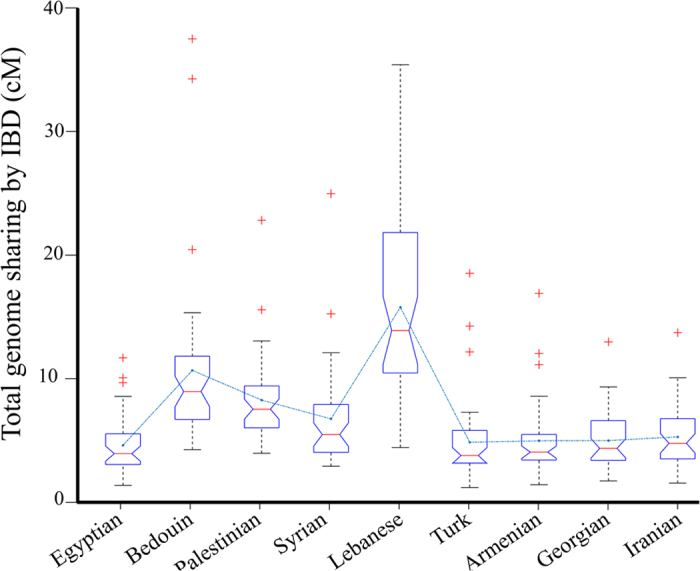
Proportion of total IBD sharing between Druze and different populations. The maximal IBD between each Druze and an individual from each population are summarised in box plots. Lines pass through the mean values.

Zidan *et al.*^15^ have studied Israeli Druze that trace all four grandparents to the same communities in Syria and Lebanon. The authors argued that the Druze are a ‘population isolate’ based on two analyses. First, a PC analysis portrayed Druze as clustered separately from “any other population” enveloped by genetic nothingness and immersed in a genetic vacuum. This statement is peculiar since not only were several populations for which genetic data were available not tested, but some of the populations included in latter analyses were excluded from this analysis, critically Lebanese, who, like Eurasian Jews, have been repeatedly shown to cluster with the Druze in other PC analyses^17^,^21^,^53^,^54^. Second, an identical-by-descent (IBD) analysis yielded no shared segments between Druze and non-Druze. However, the IBD segments used for that analysis were 3 cM, 15 times higher than the recommended threshold^55^. Had the authors applied a more reasonable threshold of 1 cM, as shown in Fig. 6, they would have likely obtained a much higher IBD sharing between the Lebanese Druze and Lebanese non-Druze, as can be expected from the Lebanese origins of the Druze included in their cohort.

Not only does the genetic isolation theory remain unsupported by prior genetic studies but strong genetic evidence exists to the contrary. The Druze inbreeding coefficient and runs of homozygosity are typical of Levantine populations, like Palestinians and Bedouins^16^,^56^, none of which have ever been considered a ‘population isolate’ on these grounds. Levantine populations showed higher inbreeding coefficients and longer runs of homozygosity compared to Africans and Europeans, but lower compared to Central Asian and American populations. These results are to be expected given the high level of consanguinity among Druze (47%), Muslim Arabs (41.7%) and Bedouins (60.1%)^57^. The Druze’s effective population size (5,700 ± 300) is much higher than would be expected for a population isolate and is within the same order of magnitude as Palestinians (7,000 ± 300) and Bedouins (6,500 ± 300)^58^.

Our results further challenge isolation perceptions on several grounds: first, the Druze admixture signature is very similar to that of neighbouring Levantine populations (Figs 1 and 4 27), indicating the existence of gene flow between them. Second, the genetic distances within Druze are at the low 20^th^ percentile (Figure S1), and none of the populations exhibiting shorter distances have been denoted a “genetic isolate”. Third, the Druze exhibit high genetic diversity (as evident from their GPS results) (Fig. 3[A1]), whereas a population isolate would be expected to be highly clustered and genetically homogenous. We have shown that the Near Eastern genetic signature of the Druze decays (Figs 1 and 3[A1,A2]) likely due to gene exchange with other Levantine populations. Finally, the Druze have a largely similar haplogroup diversity to other Levantine populations (Fig. 3).

Stereotyping populations as ‘genetic isolates’ has been criticised by geneticists and non-geneticists alike. Lipphardt^50^ demonstrated that biologists and geneticists use historical, social and administrative data to promote the notion of population isolates. While true genetic isolates are very useful in studying evolutionary, genetic and past demographic processes, their misidentification can have harmful consequences that could actively push a population towards isolation due to the stigma and discrimination such a label could entail. An extreme example of the detrimental impact of divisive research on vulnerable populations is documented in Kyllingstad’s^59^ study on the research conducted on the Scandinavian Sami people. Throughout the mid- to late-19^th^ century scientists used a range of problematic methods to argue that the Sami people were isolated from the Norwegian minority, often noting their purportedly distinct genetic ancestry. This assumption had many negative consequences for the Sami people, impacting on their territorial and political rights while contributing to the justification of wide-spread and systematic discrimination at the hands of governments. It is fundamental that any attempt at classifying a population, such as the Druze, as ‘genetic isolates’ is approached with caution and is founded on irrefutable genetic evidence as well as historical, sociological and administrative data.

### Limitations

Our study has several limitations. First, the modest sample size of the Israeli Druze may have obscured a more complex population structure that exists within and between Israeli and non-Israeli Druze communities, as noted by^12^. Second, because GPS uses the average of all an individual’s ancestors to infer geographic origin the results could reflect either the actual origin or a mid-point of many origins. We emphasise that the biogeographical analysis that relies on deriving inferences from the geographical locations of modern-day populations^10^ is inherently limited to the time these populations obtained their concurrent population structure, which may be as old as several centuries in the Middle East (see ref. 25 on how to interpret the results of biogeographical tools). This limitation presents difficulties when inferring the population history of the Druze and requires confirmation using ancient DNA from the relevant time periods. While the ancient DNA findings are in general agreement with our results (e.g., Fig. 5 and ref. 28), the ancient individuals predate the known emergence of the Druze. Therefore, further validation using ancient individuals from the first millennium is necessary to confirm our conclusions. Finally, in the absence of religious information about the Syrian and Lebanese individuals of this study we cannot exclude the possibility that they may include Druze individuals and introduce a certain bias into our interpretations.

## Conclusions

Since the emergence of the Druze at the end of the first millennium A.D., travellers, historians and anthropologists have attempted to infer their population history without successfully reaching a consensus. The biogeographical analysis localised many of the Druze to the Zagros Mountains and the mountains surrounding Lake Van and postulated that their migration path ran along a trajectory from southeast Turkey to southeast Syria. The dating analysis points to a major admixture event, which may have occurred towards the end of the Middle Ages in support of a Seljuk ancestry for the proto-Druze. Considering the genetic relatedness of Druze to ancient Near Eastern populations, our findings suggest that the habitual preference of the Druze to high mountains, which has earned them the description “mountain dwellers”, has ancient roots. A genetic analysis of the Druze in conjunction with the accumulating evidence in literature (e.g. ref. 27) dispels unsupported allegations that the Druze represent a ‘genetic isolate’. While a religious Druze minority living in remote Levantine enclaves may practice endogamy that often results in genetic abnormalities, some of which may be due to *de novo* mutations that segregate in particular families or villages (e.g. refs 12, 22 and 60), they do not represent the majority of the population and cannot be considered a ‘population isolate’ by acceptable standards. Further large scale studies would be necessary to determine the nature of these mutations.

## Methods

### Sample collection

#### Genetic source of mtDNA and Y chromosomal haplogroups

The National Geographic Society’s Genographic Project contains genetic and demographic data from over 320,000 anonymous participants who have provided written informed consent for the use of their DNA in genetic studies (https://genographic.nationalgeographic.com/). Between the years 2005 and 2012, participants were tested for either their mtDNA or Y chromosomal haplogroups. Participants tested after 2012 were genotyped on the GenoChip microarray that includes nearly 150,000 non-functional^61^ highly informative Y-chromosomal, mitochondrial, autosomal and X-chromosomal markers^62^. We accessed the Genographic Project’s database through http://geno-web.nationalgeographic.com/geno2/dist/. Our search in this database (October 2015) retrieved 27, 254 and 502 individuals who reported having at least one Lebanese, Palestinian, or Syrian parent respectively, from whom the mtDNA or Y were inherited. Haplogroup assignment was done by the Genographic Project. These data were combined with existing datasets in the literature (Tables S4 and S5). The Druze most common mtDNA and Y haplogroups were defined as haplogroups with frequency ≥5%.

#### Genetic data of Druze

Genetic data for 42 Israeli Druze genotyped on the Illumina HumanHap650K bead array were obtained from the Human Genome Diversity Panel dataset^63^. For consistency, we analysed ~94,000 autosomal SNPs that overlapped with the Genochip microarray and allowed a high GPS accuracy to be achieved^62^. PLINK (1.07) was used to test the relatedness among Druze using the –genome flag^64^. The average PiHat was 1.8% and maximum PiHat was 5.14% indicating the absence of close relatives in our data.

#### Genetic and geographic data of reference populations

To curate the reference population dataset and demonstrate the validity of our approach we obtained 1086 unrelated individuals representing 50 populations and subpopulations, with over 20 samples per population whose geographic regions were known (Table S1) from multiple studies^10^,^17^,^30^,^63^,^65^,^66^,^67^,^68^. This provided us with a comprehensive coverage of the Levant and Central Asia. For each population, we analysed a subset of 50,000–130,000 autosomal markers that overlapped with the GenoChip markers.

### Calculating the biogeographical affinity of a test sample and genetic distances

Biogeographical analyses were carried out using the Geographic Population Structure (GPS) tool as previously described^10^,^25^. GPS was shown to be highly accurate compared to alternative approaches like spatial ancestry analysis (SPA), which is, in turn, slightly more accurate than a principal component analysis (PCA) -based approach for biogeography^10^,^69^. Briefly, given the nine admixture proportions that correspond to nine putative ancestral populations of an unmixed individual, GPS converts the genetic distances between that individual and the nearest *M *= 10 reference population to geographic distances. Since GPS predictions reflect the genetic distances between the test and reference samples, further contextual interpretation of the results requires using external sources. A graph illustrating the genetic distances (*d*) was plotted using Matlab Graph function, which uses a square symmetric matrix as an adjacency matrix and constructs a weighted graph with edges corresponding to the nonzero entries of the matrix.

### Curating a reference population dataset

To infer the geographical coordinates (latitude and longitude) of an individual given *K* admixture proportions, GPS requires a reference population set of *N* populations with both *K* admixture proportions and two geographical coordinates (longitude and latitude). All supervised admixture proportions were calculated as in Elhaik *et al.*^10^.

GPS finds the biogeographical affinity of a sample by matching its admixture signature with modern-day reference samples of known biogeographical affinity then converting the genetic distances into geographic distances. For unmixed individuals, the inferred location should be interpreted as the region where populations with the most similar admixture proportions to those of the individual are found (see also ref. 25).

Detailed annotation for subpopulations was unavailable for most populations (Figure S1), though they exhibited fragmented subpopulation structure (Fig. 1). To determine the number of subpopulations in each population, we adopted a similar approach to Elhaik *et al.*’s^10^. Let *Nα* denote the number of samples per population *α*; if *Nα* was less than four individuals, the population was left unchanged. For other populations, we used *k*-means clustering routine with five replications implemented in Matlab. Let *X*_*ij*_ be the admixture proportions of individual *i* in component *j*. For each population, we ran *k*-means clustering for *k* ∈ 2, using *Nα* × 9 matrix of admixture proportions (X_*ij*_) as input. At each iteration, we calculated the ratio of the mean square and sum of squares between the groups. If this ratio was <0.9 and there were more than three samples in each cluster, then we accepted the *k*-component model, whereas smaller clusters were removed.

To bolster the accuracy of GPS inferences to beyond what has previously been reported^10^, we have updated the reference panel to comprise of highly localised Afro-Eurasian populations. For this, we applied GPS to all individuals (Table S1) using the ‘*leave-one-out*’ procedure at the population level. This approach is more rigorous than the ‘*leave-one-out*’ individual procedure and ensures that the reference panel will not be biased by outliers that do not fit with the genetic profile of the region. Individuals predicted to reside within the political borders of their countries, or less than 200 km outside of them, were retained and used to recompile the reference population set using the technique described above. We resort to the use of country labels, since some of the population data did not have detailed regional information. Elhaik *et al.*^10^ showed that there is no correlation between country size and the accuracy of the results. This procedure was repeated until the rate of correctly assigned individuals exceeded 80%. Overall, we included 39 populations, with some appearing as two subpopulations, in our reference population set, all of which were highly localised, had more than four individuals, and survived the *leave-one-out*’ procedure at the population level (Fig. 2). All these populations were considered hereafter as *reference populations*.

### Dating the admixture events

The time of admixture events were estimated with Alder v.1.02^26^ using a generation time of 25 years. We tested various combinations of Yoruba (YRI), Han Chinese (CHB), North and Central Europeans (CEU) and Italians (TSI)^70^ as reference populations, but only a YRI-TSI combination was successful and yielded statistically significant (*p*-value < 0.0001) results for the Druze.

### Ancient DNA analysis

To test the similarity between modern day individuals and ancient Anatolian, Armenian and Levantine populations, we analysed an ancient dataset that comprised of 281 ancient humans^28^. The 25 Anatolian, 17 Armenian and 22 Levantine genomes from that dataset were merged with 42 Druze, 16 Syrians, 45 Bedouins, 46 Palestinians and 8 Lebanese from the HGDP data (Table S6). A linkage disequilibrium (LD)-pruned dataset was created by removing one member of any pair of SNPs in strong LD (r^2^ > 0.2) in windows of 50 SNPs (sliding the window by 5 SNPs at a time) using indep-pairwise in PLINK^64^. The final dataset consisted of 155,486 autosomal SNPs that were used in a supervised ADMIXTURE analysis^71^.

### Identical by descent (IBD) analysis

To detect IBD segments between Druze and other populations (Table S7)^17^,^63^, we ran fastIBD 10 times using different random seeds on the entire genome-wide dataset and combined the results as described by Browning and Browning^72^. Segments were considered to be IBD only if the fastIBD score of the combined analysis was less than e^−10^. This low threshold corresponds to long shared haplotypes (≥1 cM) that are likely to be IBD. Short gaps (<50 indexes) separating long domains were assumed to be false-negatives and concatenated^72^. Pairwise-IBD segments between Druze and different populations were obtained by finding the maximum total IBD sharing between each Druze and all other individuals of a particular population.

### Maps and geographical definitions

The Nabataean caravan and trade routes map was plotted according to the map drawn by Augé and Dentzer^73^. The terms Middle East, Near East and Levant have overlapping and inconsistent meanings in the literature. Following^17^ (their Supplementary Figure 1), we defined the Levant as the area comprising Israel, Lebanon, Eastern Jordan and Syria, the Near East as the area comprising Turkey, Syria, Iraq, Lebanon, Jordan and Israel and the Middle East as the area comprising the Near East, the Arabian peninsula, Iran and south Caucasus.

## Additional Information

**How to cite this article**: Marshall, S. *et al.* Reconstructing Druze population history. *Sci. Rep.* **6**, 35837; doi: 10.1038/srep35837 (2016).

**Publisher’s note:** Springer Nature remains neutral with regard to jurisdictional claims in published maps and institutional affiliations.

## Supplementary Material

Supplementary Information

Supplementary Table S4

Supplementary Table S5

## Figures and Tables

**Table 1:** Hypotheses and geographical localities proposed to explain the origins of Druze.

Hypotheses (Geographical locality)	Description
Arabian hypothesis (Arabian Peninsula/Saudi Arabia)	Druze have descended from Arabian tribes (including Tanukhs, Kalbs, Kilabs, Tayyi and Itureans) who have migrated north from Saudi Arabia and settled in pre-Islamic Syria (Druze Heritage Foundation) prior to the 7^th^ century A.D. Many of these tribes are known to have controlled the Levantine trade routes and inhabited the mountainous regions of Syria and Lebanon^4^ together with the Druze^75^. One such tribe, the Tanukhs, are mentioned in some of the Druze Canon epistles.
Near East hypothesis (Heterogeneous Near Eastern)	The Druze have a Near Eastern-Levantine origin comprising of Turkish and Armenian migrants who cohabitated with the local populations inhabiting the Levant and the mountain regions after accepting the new faith^12^,^76^. This hypothesis is supported by an analysis of Y haplotypes, showing small genetic distances between Druze and Turks, Armenians, Iranians and finally Egyptians^12^.
Persian hypothesis (Iran and Iraq)	Hitti^4^ claimed that Druzism founders established much of their religious terminology based on Persian texts and therefore were likely Persians or Kurds who had migrated to Lebanon throughout the first century of Islamic domination. This hypothesis was criticised for underestimating the extent of Arabs living in the Levant^38^. Moreover, Sprengling^76^ argued that the Persian religious vocabulary has existed in Arabic languages since before 1000 A.D.
