# Supplementary material for: Reconstructing Druze population history
Source: Sci Rep. 2016 Nov 16;6:35837. doi: 10.1038/srep35837 (PMC5111078; doi:10.1038/srep35837)
Supplement: Supplementary Information [file srep35837-s1.pdf]

Supplementary materials

Reconstructing Druze population history

Scarlett Marshall[1], Ranajit Das[2], Mehdi Pirooznia[3] and Eran Elhaik[4*]

[1] An independent researcher

[2] Manipal Centre for Natural Sciences (MCNS), Manipal University, Manipal, Karnataka, India

[3] Johns Hopkins University, Department of Psychiatry and Behavioral Sciences, Baltimore, MD, USA 21205

[4] University of Sheffield, Department of Animal and Plant Sciences, Sheffield, UK S10 2TN

Figure S1

The genetic distances (*d*) within each reference population.

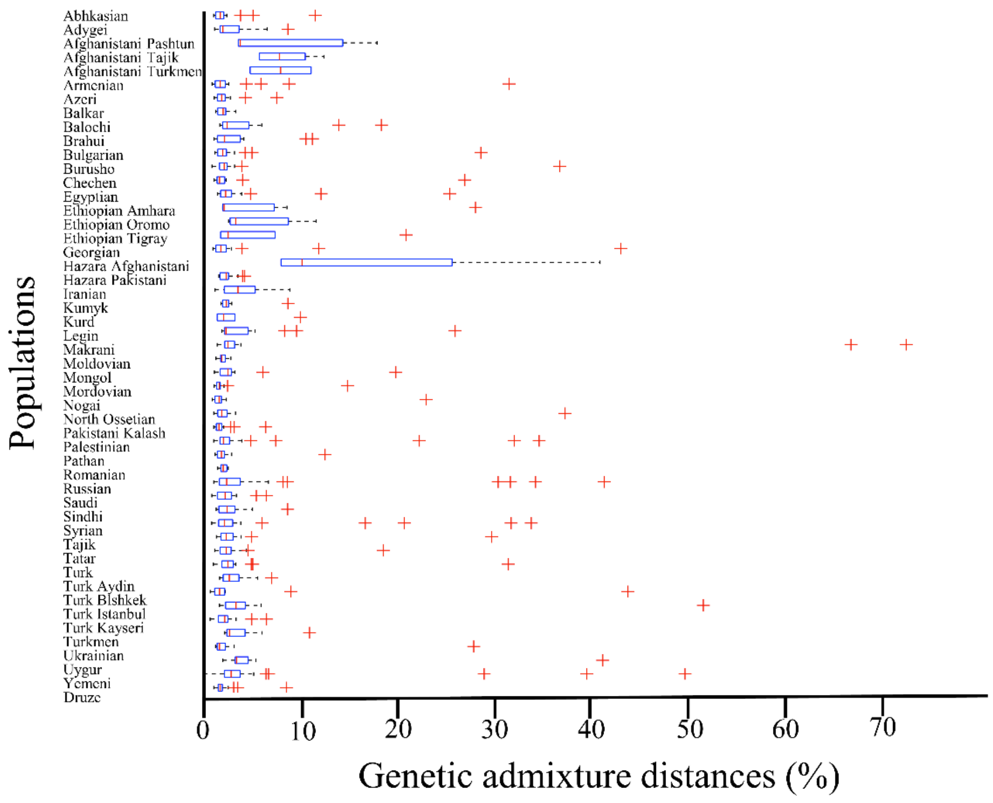

Figure S2

The genetic distances (*d*) between Druze and other populations

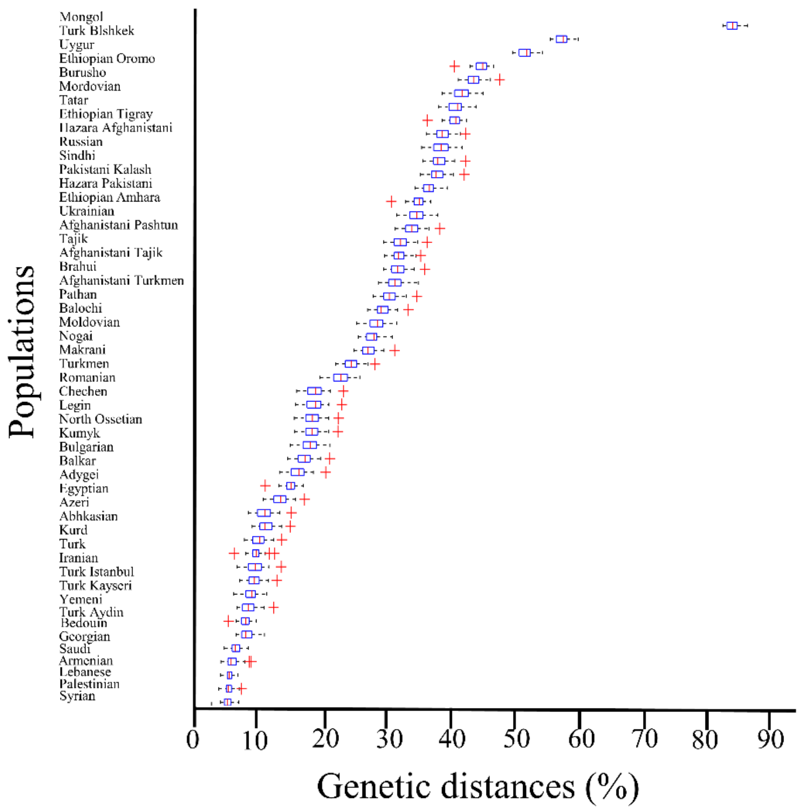

Table S1

Summary of reference populations, nicknames, sampling sites, sample sizes (*N*), geographic coordinates, and data sources used for each population

Populations	Nick	Countries and states/regions	*N*	Latitude	Longitude	Sources
Abkhazian	AB	Abkhazia	27	42.97	41.44	65, 62, 10
Afghan, Hazara	AHAZ	Afghanistan, Hazarajat	5	36.89	73.26	25
Afghan, Pashtun	AFP	Afghanistan	5	36.89	73.26	25
Afghan, Tadjik	AFTA	Afghanistan	5	36.89	73.26	25
Afghan, Turkmen	AFTU	Afghanistan	4	36.89	73.26	25
Armenian	ARM	Armenia	35	40.08	45.02	65, 62
Bedouins	BDN	Israel	45	32.25	35	59
Bulgarian	BU	Bulgaria	28	42.41	23.19	65, 10
Egyptian	EG	Egypt	27	26.82	30.8	17, 10
Ethiopian, Amhara	ETA	Ethiopia	7	9.145	40.49	17
Ethiopian, Oromo	ETO	Ethiopia	7	9.145	40.49	17
Ethiopian, Tigray	ETT	Ethiopia	5	9.145	40.49	17
Georgian	GO	Georgia	34	41.43	44.47	17, 62, 10
Iranian	IR	Iran	36	32.42	53.68	17, 10
Iranian, Azeri	IAZ	Iran, Azerbaijan	16	48	38	62
Kurd	KR	Kazakhstan	6	36.72	42.45	65
Lebanese	LEB	Lebanon	30	33.85	35.86	17, 10
Moldovian	MOL	Moldova	7	28.12	47.68	62
Mongolian	MG	Mongolia	21	45	111	10, 59
Pakistani, Balochi	PBA	Pakistan, Balochistan	25	30.18	67.01	59
Pakistani, Brahui	PBR	Pakistan, Balochistan	25	30.5	66.5	59
Pakistani, Burusho	PBU	Pakistan, Chitral, Khyber Pakhtunkhwa	25	36.5	74	59
Pakistani, Hazara	PHAZ	Pakistan, Khyber Pakhtunkhwa	23	33.5	70	59
Pakistani, Kalash	PKA	Pakistan, Chitral, Khyber Pakhtunkhwa	25	27.83	68.77	59
Pakistani, Makrani	PMA	Pakistan, Makran, Balochistan	25	26	64	59
Pakistani, Pathan	PPA	Pakistan	23	33.5	70.5	59
Pakistani, Sindhi	PSI	Pakistan, Sindh	25	25.5	69	59
Palestinian	PAL	Israel	57	32.5	35	59, 62
Romanian	RO	Romania	15	44.8	26.06	10
Russian, Adygei	RAD	Russia, Adygea	17	44	29	59
Russian, Balkar	RB	Russia, Balkar	22	43.39	43.56	65,

						62
Russian, Chechen	RC	Russia, Chechnya	24	43.4	45.71	65, 10
Russian, Kumyk	RK	Russia, Republic of Dagestan	17	42.14	47.08	65 62
Russian, Lezgin	ILZ	Russia, Republic of Dagestan	21	42	47.96	17, 62
Russian, Mordovian	RMO	Russia, Mordvinia	15	54.23	44.06	65
Russian, Moscow	RM	Russia, Moscow	39	55.75	37.62	62, 10
Russian, Nogai	RN	Russia, Republic of Dagestan	16	42.14	47.09	65
Russian, North Ossetian	RNO	Russia, Ossetia (North)	18	43.04	44.28	65, 62
Russian, Tatar	RT	Russia, Tatarstan	35	55.18	50.72	62, 10
Saudi	SDI	Saudi Arabia	20	23.88	45.07	17
Syrian	SIN	Syria	16	34.8	38.99	17
Tajik	PT	Tajikistan	28	38.35	68.48	10, 65
Turk	TUR	Turkey	19	39	36	17
Turk, Aydin	TAY	Turkey	20	37.29	28.61	64
Turk, Bishkek	TBI	Turkey	16	41.20	74.77	64
Turk, Istanbul	TIS	Turkey	20	41	28.98	64
Turk, Kayseri	TKA	Turkey	23	38.51	33.38	64
Turk, Uygur	TUY	Turkey	10	44	81	59
Turkmen	TR	Turkmenistan	23	37.57	58.23	65, 62
Ukrainian	UR	Ukraine	20	50.26	30.31	65
Yemenite	YM	Yemen	74	15.55	48.51	17, 63

Table S2

Prediction accuracy for populations and sub populations

	Prediction accuracy (%)	
**Populations**	**<250km**	**<500km**
Abkhazian	96	96
Adygei	12	82
Afghanistan Pashtun	100	100
Afghanistan Tajik	80	100
Afghanistan Turkmen	25	100
Armenian	86	86
Azeri	0	13
Balochi	100	100
Bedouin	38	42
Brahui	100	100
Bulgarian	100	100
Burusho	100	100
Egyptian	89	89
Ethiopian A	100	100
Ethiopian O	100	100
Ethiopian T	100	100
Georgian	79	82
Hazara Afghanistan	60	60
Hazara Pakistan	4	4
Iranian	56	83
Kurds	0	0
Lebanese	23	43
Lezgin	86	100
Makrani	96	96
Moldovian	14	57
Mongolian	100	100
Pakistani Kalash	100	100
Palestinian	67	95

Pathan	96	100
Romanian	93	100
Russian, Balkar	91	91
Russian, Chechen	100	100
Russian, Kumyk	71	100
Russian, Mordovian	93	100
Russian, Moscow	67	67
Russian, Nogai	6	6
Russian, North Ossetian	100	100
Russian, Tatar	0	0
Saudi	75	75
Sindhi	96	96
Syrian	94	100
Tajikistan	61	96
Turk	89	89
Turkey Aydin	65	80
Turkey Bishkek	19	44
Turkey Istanbul	80	80
Turkey Kayseri	91	91
Turkmen	87	87
Ukrainian	75	100
Uygur	100	100
Yemen	28	38

Table S3

GPS predictions of more recent and ancient origins for Druze individuals

	Recent origin		Ancient origin	
**Ind ID**	**GPS Lat**	**GPS Long**	**GPS Lat**	**GPS Long**
HGDP00557	34.72	40.54	36.50	43.56
HGDP00569	36.13	40.58	37.56	42.85
HGDP00581	36.40	41.08	38.30	44.15
HGDP00594	35.87	41.08	37.03	43.38
HGDP00558	38.04	43.29	38.65	44.26
HGDP00582	35.32	39.60	37.51	42.81
HGDP00595	36.87	41.12	38.65	42.50
HGDP00559	36.59	41.19	38.32	43.86
HGDP00571	39.38	44.40	39.70	44.86
HGDP00583	34.95	40.46	36.86	43.43
HGDP00597	35.95	41.41	37.00	43.78
HGDP00572	34.61	39.29	33.41	38.07
HGDP00584	35.56	39.75	37.28	42.38
HGDP00598	35.38	40.51	36.75	43.05
HGDP00561	34.88	39.81	36.85	42.93
HGDP00573	36.25	40.61	38.31	42.56
HGDP00599	38.26	42.94	39.28	43.85
HGDP00562	35.95	40.32	37.61	42.54
HGDP00574	38.22	43.06	38.74	44.22
HGDP00586	34.31	38.80	32.89	38.09
HGDP00600	35.43	41.16	36.67	43.76
HGDP00563	35.59	41.28	36.98	43.82
HGDP00575	36.71	40.98	38.82	42.77
HGDP00587	35.97	39.87	37.86	41.14
HGDP00601	33.84	40.20	36.11	43.68
HGDP00565	34.42	40.24	36.61	43.27
HGDP00577	34.81	39.70	33.91	38.88
HGDP00564	34.39	40.13	35.85	43.88
HGDP00576	33.77	37.70	32.18	35.67
HGDP00588	36.60	41.21	37.83	43.42
HGDP00602	36.12	41.33	37.17	43.58
HGDP00566	32.58	39.43	31.01	38.38
HGDP00578	34.12	39.31	32.27	38.46
HGDP00590	35.62	41.38	36.77	43.72
HGDP00604	38.19	43.00	39.07	43.65
HGDP00567	33.82	39.11	31.92	38.45
HGDP00579	31.78	40.48	26.53	43.25
HGDP00591	36.49	40.90	39.03	43.47
HGDP00560	38.43	43.41	39.17	44.57
HGDP00568	38.36	43.27	38.83	44.33

HGDP00580	36.05	40.19	38.08	41.86
HGDP00606	33.82	39.40	32.32	38.74

Table S6

Summary of ancient and modern individuals used in the supervised admixture analysis.

Sample ID	Population label	Source
I1584	Anatolia (Chalcolithic)	Lazaridis et al. (2016)
I0707	Anatolia (Neolithic)	Lazaridis et al. (2016)
I0708	Anatolia (Neolithic)	Lazaridis et al. (2016)
I0709	Anatolia (Neolithic)	Lazaridis et al. (2016)
I0723	Anatolia (Neolithic)	Lazaridis et al. (2016)
I0724	Anatolia (Neolithic)	Lazaridis et al. (2016)
I0726	Anatolia (Neolithic)	Lazaridis et al. (2016)
I0727	Anatolia (Neolithic)	Lazaridis et al. (2016)
I0736	Anatolia (Neolithic)	Lazaridis et al. (2016)
I0744	Anatolia (Neolithic)	Lazaridis et al. (2016)
I0745	Anatolia (Neolithic)	Lazaridis et al. (2016)
I0746	Anatolia (Neolithic)	Lazaridis et al. (2016)
I1096	Anatolia (Neolithic)	Lazaridis et al. (2016)
I1097	Anatolia (Neolithic)	Lazaridis et al. (2016)
I1098	Anatolia (Neolithic)	Lazaridis et al. (2016)
I1099	Anatolia (Neolithic)	Lazaridis et al. (2016)
I1100	Anatolia (Neolithic)	Lazaridis et al. (2016)
I1101	Anatolia (Neolithic)	Lazaridis et al. (2016)
I1102	Anatolia (Neolithic)	Lazaridis et al. (2016)
I1103	Anatolia (Neolithic)	Lazaridis et al. (2016)
I1579	Anatolia (Neolithic)	Lazaridis et al. (2016)
I1580	Anatolia (Neolithic)	Lazaridis et al. (2016)
I1581	Anatolia (Neolithic)	Lazaridis et al. (2016)
I1583	Anatolia (Neolithic)	Lazaridis et al. (2016)
I1585	Anatolia (Neolithic)	Lazaridis et al. (2016)
I1407	Armenia (Chalcolithic)	Lazaridis et al. (2016)
I1409	Armenia (Chalcolithic)	Lazaridis et al. (2016)
I1631	Armenia (Chalcolithic)	Lazaridis et al. (2016)
I1632	Armenia (Chalcolithic)	Lazaridis et al. (2016)
I1633	Armenia (Bronze Age)	Lazaridis et al. (2016)
I1634	Armenia (Chalcolithic)	Lazaridis et al. (2016)
I1635	Armenia (Bronze Age)	Lazaridis et al. (2016)
I1656	Armenia (Bronze Age)	Lazaridis et al. (2016)
I1658	Armenia (Bronze Age)	Lazaridis et al. (2016)
RISE396	Armenia (Bronze Age)	Lazaridis et al. (2016)
RISE397	Armenia (Bronze Age)	Lazaridis et al. (2016)

RISE407	Armenia (Bronze Age)	Lazaridis et al. (2016)
RISE408	Armenia (Bronze Age)	Lazaridis et al. (2016)
RISE412	Armenia (Bronze Age)	Lazaridis et al. (2016)
RISE413	Armenia (Bronze Age)	Lazaridis et al. (2016)
RISE416	Armenia (Bronze Age)	Lazaridis et al. (2016)
RISE423	Armenia (Bronze Age)	Lazaridis et al. (2016)
I0861	Levant (Natufian)	Lazaridis et al. (2016)
I0867	Levant (Neolithic)	Lazaridis et al. (2016)
I1069	Levant (Natufian)	Lazaridis et al. (2016)
I1072	Levant (Natufian)	Lazaridis et al. (2016)
I1414	Levant (Neolithic)	Lazaridis et al. (2016)
I1415	Levant (Neolithic)	Lazaridis et al. (2016)
I1416	Levant (Neolithic)	Lazaridis et al. (2016)
I1679	Levant (Neolithic)	Lazaridis et al. (2016)
I1685	Levant (Natufian)	Lazaridis et al. (2016)
I1687	Levant (Natufian)	Lazaridis et al. (2016)
I1690	Levant (Natufian)	Lazaridis et al. (2016)
I1699	Levant (Neolithic)	Lazaridis et al. (2016)
I1700	Levant (Neolithic)	Lazaridis et al. (2016)
I1701	Levant (Neolithic)	Lazaridis et al. (2016)
I1704	Levant (Neolithic)	Lazaridis et al. (2016)
I1705	Levant (Bronze Age)	Lazaridis et al. (2016)
I1706	Levant (Bronze Age)	Lazaridis et al. (2016)
I1707	Levant (Neolithic)	Lazaridis et al. (2016)
I1709	Levant (Neolithic)	Lazaridis et al. (2016)
I1710	Levant (Neolithic)	Lazaridis et al. (2016)
I1727	Levant (Neolithic)	Lazaridis et al. (2016)
I1730	Levant (Bronze Age)	Lazaridis et al. (2016)
HGDP00607	Bedouin	HGDP
HGDP00608	Bedouin	HGDP
HGDP00609	Bedouin	HGDP
HGDP00610	Bedouin	HGDP
HGDP00611	Bedouin	HGDP
HGDP00612	Bedouin	HGDP
HGDP00613	Bedouin	HGDP
HGDP00614	Bedouin	HGDP
HGDP00615	Bedouin	HGDP
HGDP00616	Bedouin	HGDP
HGDP00618	Bedouin	HGDP
HGDP00619	Bedouin	HGDP
HGDP00620	Bedouin	HGDP
HGDP00622	Bedouin	HGDP
HGDP00623	Bedouin	HGDP
HGDP00624	Bedouin	HGDP
HGDP00625	Bedouin	HGDP

HGDP00626	Bedouin	HGDP
HGDP00627	Bedouin	HGDP
HGDP00628	Bedouin	HGDP
HGDP00629	Bedouin	HGDP
HGDP00630	Bedouin	HGDP
HGDP00631	Bedouin	HGDP
HGDP00632	Bedouin	HGDP
HGDP00634	Bedouin	HGDP
HGDP00635	Bedouin	HGDP
HGDP00636	Bedouin	HGDP
HGDP00637	Bedouin	HGDP
HGDP00638	Bedouin	HGDP
HGDP00639	Bedouin	HGDP
HGDP00640	Bedouin	HGDP
HGDP00641	Bedouin	HGDP
HGDP00642	Bedouin	HGDP
HGDP00643	Bedouin	HGDP
HGDP00644	Bedouin	HGDP
HGDP00645	Bedouin	HGDP
HGDP00646	Bedouin	HGDP
HGDP00647	Bedouin	HGDP
HGDP00648	Bedouin	HGDP
HGDP00649	Bedouin	HGDP
HGDP00650	Bedouin	HGDP
HGDP00651	Bedouin	HGDP
HGDP00653	Bedouin	HGDP
HGDP00654	Bedouin	HGDP
HGDP00701	Bedouin	HGDP
HGDP00557	Druze	HGDP
HGDP00558	Druze	HGDP
HGDP00559	Druze	HGDP
HGDP00560	Druze	HGDP
HGDP00561	Druze	HGDP
HGDP00562	Druze	HGDP
HGDP00563	Druze	HGDP
HGDP00564	Druze	HGDP
HGDP00565	Druze	HGDP
HGDP00566	Druze	HGDP
HGDP00567	Druze	HGDP
HGDP00568	Druze	HGDP
HGDP00569	Druze	HGDP
HGDP00571	Druze	HGDP
HGDP00572	Druze	HGDP
HGDP00573	Druze	HGDP
HGDP00574	Druze	HGDP
HGDP00575	Druze	HGDP
HGDP00576	Druze	HGDP

HGDP00577	Druze	HGDP
HGDP00578	Druze	HGDP
HGDP00579	Druze	HGDP
HGDP00580	Druze	HGDP
HGDP00581	Druze	HGDP
HGDP00582	Druze	HGDP
HGDP00583	Druze	HGDP
HGDP00584	Druze	HGDP
HGDP00586	Druze	HGDP
HGDP00587	Druze	HGDP
HGDP00588	Druze	HGDP
HGDP00590	Druze	HGDP
HGDP00591	Druze	HGDP
HGDP00594	Druze	HGDP
HGDP00595	Druze	HGDP
HGDP00597	Druze	HGDP
HGDP00598	Druze	HGDP
HGDP00599	Druze	HGDP
HGDP00600	Druze	HGDP
HGDP00601	Druze	HGDP
HGDP00602	Druze	HGDP
HGDP00604	Druze	HGDP
HGDP00606	Druze	HGDP
Lebanon1	Lebanese	HGDP
Lebanon2	Lebanese	HGDP
Lebanon3	Lebanese	HGDP
Lebanon4	Lebanese	HGDP
Lebanon5	Lebanese	HGDP
Lebanon6	Lebanese	HGDP
Lebanon7	Lebanese	HGDP
Lebanon8	Lebanese	HGDP
HGDP00675	Palestinian	HGDP
HGDP00676	Palestinian	HGDP
HGDP00677	Palestinian	HGDP
HGDP00678	Palestinian	HGDP
HGDP00679	Palestinian	HGDP
HGDP00680	Palestinian	HGDP
HGDP00682	Palestinian	HGDP
HGDP00683	Palestinian	HGDP
HGDP00684	Palestinian	HGDP
HGDP00685	Palestinian	HGDP
HGDP00686	Palestinian	HGDP
HGDP00687	Palestinian	HGDP
HGDP00688	Palestinian	HGDP
HGDP00689	Palestinian	HGDP
HGDP00690	Palestinian	HGDP
HGDP00691	Palestinian	HGDP

HGDP00692	Palestinian	HGDP
HGDP00693	Palestinian	HGDP
HGDP00694	Palestinian	HGDP
HGDP00696	Palestinian	HGDP
HGDP00697	Palestinian	HGDP
HGDP00698	Palestinian	HGDP
HGDP00699	Palestinian	HGDP
HGDP00700	Palestinian	HGDP
HGDP00722	Palestinian	HGDP
HGDP00723	Palestinian	HGDP
HGDP00724	Palestinian	HGDP
HGDP00725	Palestinian	HGDP
HGDP00726	Palestinian	HGDP
HGDP00727	Palestinian	HGDP
HGDP00729	Palestinian	HGDP
HGDP00730	Palestinian	HGDP
HGDP00731	Palestinian	HGDP
HGDP00732	Palestinian	HGDP
HGDP00733	Palestinian	HGDP
HGDP00734	Palestinian	HGDP
HGDP00735	Palestinian	HGDP
HGDP00736	Palestinian	HGDP
HGDP00737	Palestinian	HGDP
HGDP00738	Palestinian	HGDP
HGDP00739	Palestinian	HGDP
HGDP00740	Palestinian	HGDP
HGDP00741	Palestinian	HGDP
HGDP00744	Palestinian	HGDP
HGDP00745	Palestinian	HGDP
HGDP00746	Palestinian	HGDP
syria1	Syrians	HGDP
syria2	Syrians	HGDP
syria3	Syrians	HGDP
syria4	Syrians	HGDP
syria5	Syrians	HGDP
syria6	Syrians	HGDP
syria7	Syrians	HGDP
syria8	Syrians	HGDP
syria9	Syrians	HGDP
syria10	Syrians	HGDP
syria298	Syrians	HGDP
syria361	Syrians	HGDP
syria461	Syrians	HGDP
syria464	Syrians	HGDP
syria485	Syrians	HGDP
syria520	Syrians	HGDP

Table S7

Population ID	Population	Source
iran1	Iranian	Behar et al. (2010)
iran2	Iranian	Behar et al. (2010)
iran3	Iranian	Behar et al. (2010)
iran4	Iranian	Behar et al. (2010)
iran5	Iranian	Behar et al. (2010)
iran6	Iranian	Behar et al. (2010)
iran7	Iranian	Behar et al. (2010)
iran8	Iranian	Behar et al. (2010)
iran9	Iranian	Behar et al. (2010)
iran10	Iranian	Behar et al. (2010)
iran11	Iranian	Behar et al. (2010)
iran12	Iranian	Behar et al. (2010)
iran13	Iranian	Behar et al. (2010)
iran14	Iranian	Behar et al. (2010)
iran15	Iranian	Behar et al. (2010)
iran16	Iranian	Behar et al. (2010)
iran17	Iranian	Behar et al. (2010)
iran18	Iranian	Behar et al. (2010)
iran19	Iranian	Behar et al. (2010)
iran20	Iranian	Behar et al. (2010)
HGDP00611	Bedouin	HGDP
HGDP00623	Bedouin	HGDP
HGDP00634	Bedouin	HGDP
HGDP00645	Bedouin	HGDP
HGDP00612	Bedouin	HGDP
HGDP00624	Bedouin	HGDP
HGDP00635	Bedouin	HGDP
HGDP00646	Bedouin	HGDP
HGDP00613	Bedouin	HGDP
HGDP00636	Bedouin	HGDP
HGDP00647	Bedouin	HGDP
HGDP00614	Bedouin	HGDP
HGDP00626	Bedouin	HGDP
HGDP00637	Bedouin	HGDP
HGDP00648	Bedouin	HGDP
HGDP00615	Bedouin	HGDP
HGDP00627	Bedouin	HGDP
HGDP00638	Bedouin	HGDP
HGDP00649	Bedouin	HGDP
HGDP00616	Bedouin	HGDP
HGDP00628	Bedouin	HGDP
HGDP00639	Bedouin	HGDP
HGDP00650	Bedouin	HGDP
HGDP00629	Bedouin	HGDP

HGDP00640	Bedouin	HGDP
HGDP00651	Bedouin	HGDP
HGDP00607	Bedouin	HGDP
HGDP00619	Bedouin	HGDP
HGDP00631	Bedouin	HGDP
HGDP00642	Bedouin	HGDP
HGDP00653	Bedouin	HGDP
HGDP00641	Bedouin	HGDP
HGDP00643	Bedouin	HGDP
HGDP00654	Bedouin	HGDP
HGDP00644	Bedouin	HGDP
HGDP00701	Bedouin	HGDP
HGDP00610	Bedouin	HGDP
HGDP00622	Bedouin	HGDP
HGDP00625	Bedouin	HGDP
HGDP00608	Bedouin	HGDP
HGDP00620	Bedouin	HGDP
HGDP00632	Bedouin	HGDP
HGDP00618	Bedouin	HGDP
HGDP00630	Bedouin	HGDP
HGDP00609	Bedouin	HGDP
Egypt1	Egyptian	Behar et al. (2010)
Egypt2	Egyptian	Behar et al. (2010)
Egypt3	Egyptian	Behar et al. (2010)
Egypt4	Egyptian	Behar et al. (2010)
Egypt5	Egyptian	Behar et al. (2010)
Egypt6	Egyptian	Behar et al. (2010)
Egypt7	Egyptian	Behar et al. (2010)
Egypt8	Egyptian	Behar et al. (2010)
Egypt9	Egyptian	Behar et al. (2010)
Egypt10	Egyptian	Behar et al. (2010)
Egypt11	Egyptian	Behar et al. (2010)
Egypt12	Egyptian	Behar et al. (2010)
Jordan444	Jordanian	Behar et al. (2010)
Jordan608	Jordanian	Behar et al. (2010)
Jordan307	Jordanian	Behar et al. (2010)
Jordan646	Jordanian	Behar et al. (2010)
Jordan543	Jordanian	Behar et al. (2010)
Jordan214	Jordanian	Behar et al. (2010)
Jordan503	Jordanian	Behar et al. (2010)
Jordan384	Jordanian	Behar et al. (2010)
Jordan305	Jordanian	Behar et al. (2010)
Jordan445	Jordanian	Behar et al. (2010)
Jordan387	Jordanian	Behar et al. (2010)
Jordan62	Jordanian	Behar et al. (2010)
Jordan382	Jordanian	Behar et al. (2010)
Jordan603	Jordanian	Behar et al. (2010)

Jordan426	Jordanian	Behar et al. (2010)
Jordan502	Jordanian	Behar et al. (2010)
Jordan485	Jordanian	Behar et al. (2010)
Jordan546	Jordanian	Behar et al. (2010)
Jordan563	Jordanian	Behar et al. (2010)
Jordan560	Jordanian	Behar et al. (2010)
HGDP00693	Palestinian	HGDP
HGDP00683	Palestinian	HGDP
HGDP00682	Palestinian	HGDP
HGDP00694	Palestinian	HGDP
HGDP00725	Palestinian	HGDP
HGDP00736	Palestinian	HGDP
HGDP00684	Palestinian	HGDP
HGDP00696	Palestinian	HGDP
HGDP00727	Palestinian	HGDP
HGDP00738	Palestinian	HGDP
HGDP00685	Palestinian	HGDP
HGDP00739	Palestinian	HGDP
HGDP00686	Palestinian	HGDP
HGDP00699	Palestinian	HGDP
HGDP00731	Palestinian	HGDP
HGDP00700	Palestinian	HGDP
HGDP00732	Palestinian	HGDP
HGDP00744	Palestinian	HGDP
HGDP00722	Palestinian	HGDP
HGDP00733	Palestinian	HGDP
HGDP00745	Palestinian	HGDP
HGDP00723	Palestinian	HGDP
HGDP00734	Palestinian	HGDP
HGDP00746	Palestinian	HGDP
HGDP00724	Palestinian	HGDP
HGDP00735	Palestinian	HGDP
HGDP00740	Palestinian	HGDP
HGDP00726	Palestinian	HGDP
HGDP00737	Palestinian	HGDP
tur52	Turk	Behar et al. (2010)
tur67	Turk	Behar et al. (2010)
tur84	Turk	Behar et al. (2010)
tur110	Turk	Behar et al. (2010)
tur124	Turk	Behar et al. (2010)
tur139	Turk	Behar et al. (2010)
tur154	Turk	Behar et al. (2010)
tur170	Turk	Behar et al. (2010)
tur182	Turk	Behar et al. (2010)
tur197	Turk	Behar et al. (2010)
tur210	Turk	Behar et al. (2010)
tur222	Turk	Behar et al. (2010)

tur236	Turk	Behar et al. (2010)
tur262	Turk	Behar et al. (2010)
tur277	Turk	Behar et al. (2010)
tur306	Turk	Behar et al. (2010)
tur2	Turk	Behar et al. (2010)
tur20	Turk	Behar et al. (2010)
tur37	Turk	Behar et al. (2010)
arm3	Armenian	Behar et al. (2010)
arm12	Armenian	Behar et al. (2010)
arm21	Armenian	Behar et al. (2010)
arm4	Armenian	Behar et al. (2010)
arm13	Armenian	Behar et al. (2010)
arm23	Armenian	Behar et al. (2010)
arm5	Armenian	Behar et al. (2010)
arm6	Armenian	Behar et al. (2010)
arm26	Armenian	Behar et al. (2010)
arm7	Armenian	Behar et al. (2010)
arm17	Armenian	Behar et al. (2010)
arm8	Armenian	Behar et al. (2010)
arm14	Armenian	Behar et al. (2010)
arm9	Armenian	Behar et al. (2010)
arm18	Armenian	Behar et al. (2010)
arm10	Armenian	Behar et al. (2010)
arm19	Armenian	Behar et al. (2010)
arm11	Armenian	Behar et al. (2010)
arm20	Armenian	Behar et al. (2010)
HGDP00557	Druze	HGDP
HGDP00569	Druze	HGDP
HGDP00581	Druze	HGDP
HGDP00594	Druze	HGDP
HGDP00558	Druze	HGDP
HGDP00582	Druze	HGDP
HGDP00595	Druze	HGDP
HGDP00559	Druze	HGDP
HGDP00571	Druze	HGDP
HGDP00583	Druze	HGDP
HGDP00597	Druze	HGDP
HGDP00572	Druze	HGDP
HGDP00584	Druze	HGDP
HGDP00598	Druze	HGDP
HGDP00561	Druze	HGDP
HGDP00573	Druze	HGDP
HGDP00599	Druze	HGDP
HGDP00562	Druze	HGDP
HGDP00574	Druze	HGDP
HGDP00586	Druze	HGDP
HGDP00600	Druze	HGDP

HGDP00563	Druze	HGDP
HGDP00575	Druze	HGDP
HGDP00587	Druze	HGDP
HGDP00601	Druze	HGDP
HGDP00565	Druze	HGDP
HGDP00577	Druze	HGDP
HGDP00564	Druze	HGDP
HGDP00576	Druze	HGDP
HGDP00588	Druze	HGDP
HGDP00602	Druze	HGDP
HGDP00566	Druze	HGDP
HGDP00578	Druze	HGDP
HGDP00590	Druze	HGDP
HGDP00604	Druze	HGDP
HGDP00567	Druze	HGDP
HGDP00579	Druze	HGDP
HGDP00591	Druze	HGDP
HGDP00560	Druze	HGDP
HGDP00568	Druze	HGDP
HGDP00580	Druze	HGDP
HGDP00606	Druze	HGDP
mg65	Georgian	Behar et al. (2010)
mg20	Georgian	Behar et al. (2010)
mg43	Georgian	Behar et al. (2010)
mg5	Georgian	Behar et al. (2010)
mg34	Georgian	Behar et al. (2010)
mg68	Georgian	Behar et al. (2010)
mg54	Georgian	Behar et al. (2010)
mg64	Georgian	Behar et al. (2010)
mg22	Georgian	Behar et al. (2010)
mg47	Georgian	Behar et al. (2010)
mg27	Georgian	Behar et al. (2010)
mg23	Georgian	Behar et al. (2010)
mg49	Georgian	Behar et al. (2010)
mg40	Georgian	Behar et al. (2010)
mg51	Georgian	Behar et al. (2010)
mg62	Georgian	Behar et al. (2010)
mg31	Georgian	Behar et al. (2010)
mg61	Georgian	Behar et al. (2010)
mg70	Georgian	Behar et al. (2010)
mg72	Georgian	Behar et al. (2010)
HGDP00675	Palestinian	HGDP
HGDP00687	Palestinian	HGDP
HGDP00697	Palestinian	HGDP
HGDP00729	Palestinian	HGDP
HGDP00741	Palestinian	HGDP
HGDP00676	Palestinian	HGDP

HGDP00688	Palestinian	HGDP
HGDP00698	Palestinian	HGDP
HGDP00730	Palestinian	HGDP
HGDP00677	Palestinian	HGDP
HGDP00689	Palestinian	HGDP
HGDP00678	Palestinian	HGDP
HGDP00690	Palestinian	HGDP
HGDP00679	Palestinian	HGDP
HGDP00691	Palestinian	HGDP
HGDP00680	Palestinian	HGDP
HGDP00692	Palestinian	HGDP

Supplementary Dataset File 1
Y chromosomal haplogroup counts

Supplementary Dataset File 2
MtDNA chromosomal haplogroup counts
